# The association between body fatness and prevalent MGUS in the U.S. general population

**DOI:** 10.1016/j.canep.2026.103021

**Published:** 2026-02-16

**Authors:** Mengmeng Ji, John H. Huber, Mei Wang, Tuo Lan, Graham A. Colditz, Shi-Yi Wang, Su-Hsin Chang

**Affiliations:** a Division of Public Health Sciences, Department of Surgery, Washington University School of Medicine, St. Louis, MO, United States; b Massachusetts General Hospital, Harvard Medical School, Boston, MA, United States; c Yale School of Public Health, Yale University, New Heaven, CT, United States

**Keywords:** MGUS, Obesity, BMI, Body fat percentage

## Abstract

**Objective:**

Current evidence regarding the association between obesity and monoclonal gammopathy of undetermined significance (MGUS) remains inconsistent. This study aims to evaluate the relationship between objectively measured obesity markers and prevalent MGUS using nationally representative data from the U.S. population.

**Method:**

Data came from the third National Health and Nutrition Examination Survey III (1988–1994) and continuous NHANES (1999–2004). We estimated multivariable-adjusted odds ratios (aORs) and 95 % confidence intervals (CIs) for the association between MGUS and seven obesity markers (i.e., baseline body mass index (BMI), maximum lifetime BMI, waist circumference (WC), waist-hip ratio (WHR), total body fat, fat-free mass, and body fat percentage) using logistic regression. Body composition was assessed using dual-energy X-ray absorptiometry (DXA) in NHANES 1999–2004 and tetrapolar bioelectrical impedance analysis (BIA) in NHANES III.

**Results:**

The study included 200 participants with MGUS in NHANES III (1988–1994) and 164 with MGUS in NHANES 1999–2004, compared with 12,043 participants without MGUS. In multivariate logistic regression analysis with DXA measurements, each 1 %age point increase in body fat percentage was associated with a 4 % higher odds of MGUS (aOR: 1.04, 95 % CI [1.01, 1.07]) and a 6 % higher odds of non-IgM MGUS (aOR: 1.06, 95 % CI [1.02, 1.10]). No statistically significant associations were found between MGUS and other obesity markers, including baseline BMI, maximum lifetime BMI, WC, WHR, and fat-free mass.

**Conclusion:**

Our findings indicate that obesity is associated with an increased odds of MGUS. However, many obesity markers, including the commonly used BMI, do not adequately capture this association.

## 1. Introduction

Monoclonal gammopathy of undetermined significance (MGUS) is an asymptomatic plasma cell disorder that overproduces serum monoclonal protein and could progress to more advanced diseases, such as multiple myeloma, a fatal cancer of plasma cells [1,2]. MGUS is one of the most prevalent premalignant conditions [3,4]; however, due to its asymptomatic nature, research on risk factors for MGUS is limited [5]. Existing evidence suggests that older age, male sex, black race, prior infections and inflammatory conditions, and a family history of a plasma cell malignancy increase the risk of developing MGUS [4–6].

Obesity is one of the few modifiable risk factors for multiple myeloma [7] and has been linked in the progression of MGUS to myeloma [8,9]. Nonetheless, findings on the association between obesity and MGUS remain inconsistent [5,10,11]. Two population-based studies reported no evidence for the association between obesity and MGUS [12,13]. Thordardottir *et al*. found that obesity, as defined by 11 measures, was not associated with an increased risk of MGUS among Icelandic residents [12]. An U.K study using a large primary-care database also reported no evidence for the association between high body mass index (BMI) and increased risk of developing MGUS [14]. However, two U.S. studies reported positive association between obesity and MGUS [15,16]. Using data from the Southern Community Cohort Study, Landgren *et al*. found that self-reported BMI≥ 30 kg/m^2^ were independently associated with an 80 % higher excess risk of MGUS among women [15]. A more recent study of 2628 participants who are at high risk of multiple myeloma in the PROMISE trial showed that self-reported BMI≥ 30 kg/m^2^ was positively associated with mass spectrometry-detected MGUS [16]. The discrepancy between the European and U.S. studies may be attributed to differences in participant selection, the use of subjective anthropometric measurements, and the use of varying obesity markers.

This study aims to evaluate the association between objectively measured obesity markers and the risk of developing MGUS. Previous studies that have primarily relied on self-reported anthropometric measures or based on clinical cohorts with limited generalizability. This study leverages NHANES data, a nationally representative dataset with objective body composition assessments. The findings of this study could advance our understanding of the association between obesity and MGUS in the U.S. general population. These findings will provide insights for potential screening programs for MGUS and malignant plasma cell dyscrasia or lymphoproliferative disorder.

## 2. Method

### 2.1. Data and study population

We used data from the National Health and Nutrition Examination Survey (NHANES) III, 1988–1994 and continuous NHANES 1999–2000, 2001–2002, and 2003–2004, where MGUS data are available. NHANES used a stratified multistage probability cluster design to draw samples representative of the civilian, non-institutionalized U.S. population [17, 18]. The NHANES survey combines household interviews and physical examinations, including physical and physiological measurements, blood collection and laboratory tests.

The NHANES was approved by the National Center for Health Statistics (NCHS) Research Ethics Review Board, and all participants provided written informed consent. The present study analyzed de-identified, publicly accessible NHANES data, and in accordance with federal regulations, was determined to be exempt from human subjects review. The data were accessed on April 24, 2024, and analyses were conducted through September 2024. The investigators did not have access to any information that could directly identify individual participants during or after data collection.

### 2.2. MGUS diagnosis

Testing for the presence of MGUS in NHANES was performed at the Protein Immunology Laboratory at Mayo Clinic, Rochester, Minnesota [3,19]. First, serum samples were analyzed for all identified subjects by conventional agarose-gel electrophoresis to reveal the occurrence and pattern of monoclonal protein in the study cohort. Samples with an equivocal or definite M-protein present on electrophoresis were then subjected to serum protein immunofixation, and to serum-free light--chain assay for confirmation and typing of the M-protein. Overall 6560 participants≥ 50 years of age in NHANES III and 5847 in NHANES 1999–2004 were screened for MGUS using serum samples.

### 2.3. Obesity markers

Participants were weighed in mobile examination centers, wearing only underclothing and an examination gown. Weight was recorded on a digital scale in kilograms. Standing height was measured using a stadiometer with a fixed vertical backboard and an adjustable headpiece. Waist circumference (WC) was measured just above the iliac crest using a steel measuring tape. The NHANES anthropometry procedures manual provides detailed descriptions of the measurement protocol, equipment, and quality control [20]. BMI was calculated as measured weight in kilogram (kg) divided by squared height in meter (m). The classifications were underweight (<18.5 kg/m^2^), normal weight (18.5–24.9 kg/m^2^), overweight (25.0–29.9 kg/m^2^), and obese (≥30.0 kg/m^2^). Abdominal obesity was defined as a WC of ≥ 102 cm for men and ≥ 88 cm for women. In addition, maximum lifetime BMI was constructed by using self-reported maximum lifetime weight (based on an survey question) and objectively measured height.

Body composition, including total body fat (TBF) in kg, fat-free mass (FFM) in kg, percentage body fat (%BF) was measured for adults aged ≥ 18 years (pregnant females excluded) by using dual energy X ray absorptiometry (DXA) in NHANES 1999–2004 [21]. About 22 % of subjects in the NHANES 1999–2004 had ≥ 1 missing regional body fat measurement that was due to invalid DXA scanning [22]. Because DXA data missingness is related to age, BMI, weight and height, and possibly other characteristics, participants with missing data cannot be treated as a random subset of the original sample [23]. For this reason, 5 imputed data sets for missing DXA regional body-composition measurements were generated by the NHANES 1999–2004 [22,23] and were used in this study. These imputed datasets were generated using a sequential regression multivariate imputation approach, incorporating demographic characteristics, anthropometric measures (including BMI and waist circumference), and examination-related variables [22].

In the NHANES III, participants aged ≥ 12 years (pregnant females excluded) had a single, tetrapolar bioelectrical impedance analysis measurement of resistance and reactance at 50 kHz taken between the right wrist and ankle while in a supine position, using Valhalla 1990B Bio-Resistance Body Composition Analyzer (Valhalla Scientific, San Diego, CA, USA) [24]. Following previous studies [25–27], the established equations listed below were applied to the respective NHANES III anthropometric and converted bioelectrical impedance analysis resistance data to derive estimates for FFM.

$$\text{Males}:\;\text{FFM}=-10.678+0.262*\text{Weight}+0.652*({\text{S}}^{2}/\text{Resistance})+0.015*\text{Resistance}\;\left({\text{r}}^{2}=0.90,\text{RMSE}=3.9\;\text{kg}\right),$$

$$\text{Females}:\text{FFM}=-9.529+0.168*\text{Weight}+0.696*({\text{S}}^{2}/\text{Resistance})+0.016*\text{Resistance}\;\left({\text{r}}^{2}=0.83,\text{RMSE}=2.9\;\text{kg}\right),$$

where S refers to stature measured in centimeters (cm).

Estimates for TBF and %BF for each NHANES III participant were derived from their corresponding estimated FFM using the equations: TBF = weight−FFM; %BF = TBF/weight.

### 2.4. Covariates

Covariates in the analysis included age at MGUS screening, gender (male, female), race/ethnicity (non-Hispanic White, non-Hispanic Black, Mexican American, and other race), educational attainment (less than 9th grade education, 9–11th grade education, high school, some college or associates degree, college or higher), and income-to-poverty ratio (low: <1.85, middle: 1.85–3.5, high: >3.5).

### 2.5. Statistical analysis

All analyses were accounted for the complex sampling of the NHANES, following the analytic guidelines [28]. Descriptive statistics (means and standard deviations for continuous variables and percentages for categorical variables) were calculated for demographics and obesity markers for participants and the estimated populations with and without MGUS. Independent *t*-tests were conducted for continuous variables, and Chi-square tests were used for categorical variables.

Multivariable-adjusted odds ratios (aOR) and 95 % confidence intervals (CIs) for the association between prevalent MGUS and each of the 7 obesity markers (i.e., measured BMI, maximum lifetime BMI, WC, WHR, FFM, TBF, %BF) were estimated using logistic regression. Considering the high correlation between obesity markers, as shown in Supplementary Figure S1, separate models were conducted for each marker to avoid multicollinearity. A separate subgroup analysis was conducted for patients with immunoglobulin (Ig) G, IgA or light chain MGUS, since IgM MGUS typically progresses to lymphoma and rarely to myeloma [31,32].

For the 5 imputed datasets of body composition in NHANES 1999–2004, we analyzed each dataset separately and then combine the estimates and standard errors based on Rubin’s rules for repeated-imputation inference [29]. Briefly, the combined estimate of the regression coefficient (*Q*) was the mean of the 5 individual estimates $\overset{‾}{(Q}=\sum_{i=1}^{5}{\hat{Q}}_{i}/5)$. The combined standard error for $\overset{‾}{Q}$ was calculated based on the within-imputation variance $\left(W=\sum_{i=1}^{5}{\hat{V}}_{i}/5\right)$ and the between-imputation variance $\left(B=\sum_{i=1}^{5}{\left({\hat{Q}}_{i}-\overset{‾}{Q}\right)}^{2}/4\right)$, where ${\hat{V}}_{i}$ de-notes the variance estimates across the 5 imputed datasets. The total variance combines the within- and between-imputation variances $\left(T=W+\frac{6}{5}*B\right)$. The details were described in the technical documentation by NCHS [30].

All tests were two-sided, and p-values < 0.05 were considered to be statistically significant. All analyses were conducted with in STATA 17 SE version (StataCorp, College Station, TX).

## 3. Results

A total of 364 participants tested positive for MGUS (200 MGUS cases in NHANES III and 164 in NHANES 1999–2004), representing the size of the U.S. population with MGUS of 1493,412, while 12,043 participants tested negative for MGUS, representing the size of the U.S. population without MGUS of 60,992,474 (see Table 1). The prevalence of MGUS among the U.S. population age 50 years or older during 1999–2004 was 2.3 % (95 % confidence interval [CI]: 1.8–2.8 %). Among the MGUS population, 9.66 % were IgA, 69.66 % were IgG, 12.80 % were IgM, and 7.88 % were biclonal. Populations with MGUS were more likely to be older (MGUS: 69.23 vs. No MGUS: 64.07 years; P < 0.001) and male (54.55 % vs. 45.26 %; P = 0.011) than those without MGUS. There was a higher percentage of non-Hispanic Black participants in the MGUS group (13.06 % vs 8.06 %; P = 0.028). No statistically significant differences were found in educational attainment or poverty income ratio between the two populations. The mean BMI at screening was similar between the two populations (28.14 vs 27.96 kg/m^2^; P = 0.606), and there were no statistically significant differences in BMI categories. Maximum lifetime BMI and WC were also comparable between the two populations (P = 0.363 and P = 0.210, respectively). However, the WHR was statistically significantly different and higher in the population with MGUS than in the population without MGUS (0.97 vs 0.95; P = 0.032).

In multivariable logistic regression analyses restricted to participants in NHANES 1999–2004, in whom body fat percentage was assessed using DXA, each 1 %age point increase in body fat percentage was associated with a 4 % (aOR: 1.04, 95 % CI [1.01. 1.07]) higher odds of MGUS (Table 2). Each 1 %age point increase in body fat percentage was associated with a 6 % (aOR: 1.06, 95 % CI [1.02. 1.10]) higher odds of non-IgM MGUS (Table 3). In contrast, body fat assessed using BIA was not significantly associated with prevalent MGUS. No statistically significant association was found between each of the other obesity markers (i.e., measured BMI, maximum lifetime BMI, WC, WHR, and fat free mass) and MGUS. It is noted that obesity (BMI ≥30 kg/m^2^), compared with normal weight (BMI 18.5–24.9 kg/m^2^), showed a trend toward higher odds of MGUS (P = 0.061) and IgG/IgA MGUS (P = 0.070). The association between body composition and prevalent MGUS using the 5 imputed data from NHANES were consistent with the main analyses (Supplementary Table S1).

## 4. Discussion

This study is the first to utilize nationally representative data to investigate the association between obesity markers other than BMI and prevalent MGUS in the U.S. population aged 50 years or older. Our findings indicate that a 1 % increase in body fat percentage is associated with a 4 % increase in the odds of MGUS. However, other obesity markers, including the widely used BMI, showed no statistically significant association with MGUS prevalence.

Our results suggest that the inconsistent findings from previous studies may be caused by using different obesity measures [3,15]. While most prior studies have relied primarily on BMI to examine associations between obesity and MGUS, Thordardottir et al. evaluated multiple adiposity measures, including body composition, and observed no association between obesity and MGUS among residents of Reykjavik [12]. Differences in study populations may partly explain these discrepant findings, as the Reykjavik Study was conducted in a relatively homogeneous Icelandic population, whereas our analysis was based on a nationally representative and more heterogeneous U.S. population.

BMI is a convenient and commonly used metric for obesity in epidemiological studies, but it is a limited proxy for fat mass and distribution [33,34]. Of note, the Pearson correlation coefficient between BMI and percentage body fat was only 0.58 in NHANES participants. Compared to BMI, bioelectrical impedance analysis and digital anthropometry both have the potential to provide accurate measures of fat mass and distribution in clinical settings. This study suggests that monitoring body fat percentage using advanced digital anthropometry methods, such as DXA, provides more accurate and informative assessments than bioelectrical impedance analysis for identifying individuals at higher risk of MGUS. Nonetheless, the measurement of body fat percentage may be influenced by within-lab variability and within-person variability. Within-lab variability may arise from differences in equipment or operators. Further evaluation of the impact of this variability on the results is necessary. Additionally, within-person variability may introduce errors due to changes in measurement timing, diet, or physical activity levels. Future studies could consider using the average across multiple measurements of body fat percentage per individual to reduce the impact of this variability.

The mechanism by which increased fat percentage elevates the risk of MGUS may be multifactorial, involving various metabolic and inflammatory pathways [35]. Adipose tissue is not merely a passive storage depot for excess energy but an active endocrine organ that secretes numerous bioactive molecules known as adipokines. These adipokines, including leptin, adiponectin, and pro-inflammatory cytokines, can create a chronic low-grade inflammatory state [36,37]. And chronic inflammation is a well-known risk factor for the development of various malignancies, including hematologic cancers [37–39]. Additionally, increased adiposity is associated with higher levels of insulin-like growth factor, creating a pro-oncogenic environment that may contribute to the initiation and progression of plasma cell disorders [40]. Animal studies also have shown that diet-induced obesity is associated with the development of MGUS in wild-type mice, linked to increased levels of insulin-like growth factor [41].

Our findings highlight an association between body fat percentage and prevalent MGUS, suggesting that maintenance of a healthy body fat profile may be important, independent of body weight and weight loss. Lifestyle factors related to body composition may be relevant to the development of MGUS; however, evidence on specific modifiable behaviors remains limited. General dietary patterns associated with healthier body composition, such as balanced diets emphasizing fruits, vegetables, and whole grains and limiting highly processed foods, have been linked to lower adiposity in prior studies [42], but their direct relationship with MGUS has not been well established. Similarly, physical activity, particularly resistance and strength-based exercise, may contribute to maintenance of lean mass and favorable body fat distribution, which could be relevant to MGUS prevalence, although data specific to MGUS are sparse. While higher levels of physical activity have been inversely associated with MGUS among a high-risk population [16], these findings should be interpreted cautiously, and further longitudinal research is needed to clarify whether and how lifestyle-related factors influencing body composition may relate to MGUS development.

This study leverages the NHANES, which provides nationally representative data, enhancing the generalizability of our findings. Unlike many studies that rely solely on self-reported BMI, our analysis incorporates seven obesity markers, six of which were objectively measured. This approach allows for a more nuanced and accurate assessment of the relationship between various aspects of obesity and the risk of MGUS. The comprehensive analysis of multiple obesity markers offers valuable insights into how different measures of obesity contribute to the risk of developing MGUS.

Several limitations should also be noted. Given anthropometric measurements and blood samples were collected contemporaneously as part of the NHANES examination, the observed associations should not be interpreted as evidence that obesity increases the risk of developing MGUS, but rather as associations with the prevalence or odds of MGUS, and should be interpreted with caution. In addition, NHANES does not provide detailed body composition measures distinguishing visceral and subcutaneous adipose tissue, which may have differential biological relevance for MGUS development. The relatively small number of MGUS cases limited our ability to conduct subgroup or effect-modification analyses by sex or race, which may mask potential heterogeneity in associations across population subgroups. A substantial proportion of DXA-based body composition data in NHANES 1999–2004 were missing and addressed using multiple imputation. Although the NHANES imputation strategy was designed to account for systematic missingness related to body size and other participant characteristics, imputed body fat percentage, particularly among individuals with missing DXA scans, may partially reflect information contained in correlated anthropometric measures such as BMI and waist circumference. As a result, residual bias and attenuation of distinctions between body composition and conventional obesity metrics cannot be fully excluded. As with all observational studies, there is the potential for confounding factors that could affect the validity of our findings. Despite statistical adjustments to account for known confounders, residual confounding may still influence the results. Further prospective studies are needed to confirm these associations and to explore the underlying mechanisms between obesity and MGUS.

## 5. Conclusion

This study utilized nationally representative data and found a positive association between fat percentage and the risk of developing MGUS. Our findings highlight that many obesity markers, including the commonly used BMI, do not adequately capture this association. This underscores the need for employing a range of objectively measured obesity markers to fully understand the obesity-MGUS relationship. While our results broaden the understanding of the association of potential obesity markers with MGUS, the retrospective nature of the data limits our ability to infer causality. Future studies are essential to confirm these findings and elucidate the mechanisms by which obesity may influence MGUS risk.

## Supplementary Material

1

## Figures and Tables

**Table 1:** Characteristics of the study participants from NHANES III and NHANES 1999–2004.

	With MGUS n = 364 (N = 1493,412)	Without MGUS n = 12,043 (N = 60,992,474)
NHANES period, n		
NHANES III	200	6360
NHANES 1999–2004	164	5683
Obesity Markers		
BMI at screening, mean (SE)	28.14 (0.34)	27.96 (0.09)
BMI categories, % (SE)		
Normal: 18.5–25	25.28 (2.74)	30.11 (0.71)
Underweight: ≤ 18.5	1.72 (0.83)	1.36 (0.15)
Overweight: 25.0 to < 30	40.16 (2.90)	37.30 (0.55)
Obese: ≥ 30	30.79 (3.00)	29.40 (0.63)
Maximum lifetime BMI^a^, mean (SE)	31.19 (0.59)	30.66 (0.15)
Waist circumference (cm), mean (SE)	99.73 (0.99)	98.46 (0.22)
Waist categories, % (SE)		
Normal risk (men ≤102 cm; women ≤88 cm)	42.04 (3.63)	40.92 (0.80)
High risk (men >102 cm; women >88 cm)	57.96 (3.63)	59.08 (0.80)
Waist to hip ratio^b^	0.97 (0.01)	0.95 (0.002)
Fat-free mass (DXA)^a^, kg	51.69 (11.42)	50.41 (12.03)
Fat-free mass (BIA)^b^, kg	52.39 (12.17)	50.22 (11.43)
Total body fat (DXA)^a^, kg	28.73 (10.26)	29.01 (10.54)
Total body fat (BIA)^b^, kg	22.66 (9.69)	24.54 (9.77)
Body percentage fat (DXA)^a^, %	35.40 (7.69)	36.14 (8.13)
Body percentage fat (BIA)^b^, %	29.60 (8.87)	32.35 (8.74)
MGUS Ig isotype, n (%)		
IgA	26 (9.66 %)	/
IgG	259 (69.66 %)	/
IgM	44 (12.80 %)	/
biclonal	35 (7.88 %)	/
Age in years, mean (SE)	69.23 (0.68)	64.07 (0.21)
Gender, % (SE)		
Male	54.55 (3.3)	45.26 (0.49)
Female	45.45 (3.3)	54.74 (0.49)
Race/ethnicity, % (SE)		
Non-Hispanic White	79.67 (2.86)	81.43 (1.29)
Non-Hispanic Black	13.06 (1.75)	8.06 (0.57)
Hispanic	2.4 (0.6)	3.2 (0.43)
Other	4.88 (1.79)	7.31 (0.96)
Education, % (SE)		
< 9th grade	15.73 (2.65)	15.25 (0.64)
9–11th grade	16.16 (2.72)	14.43 (0.62)
High school	28.09 (3.12)	29.24 (0.68)
Some college	20.61 (2.54)	20.86 (0.62)
College graduate or higher	19.41 (2.22)	20.21 (0.82)
Poverty income ratio, % (SE)		
Low (<1.85)	26.48 (2.71)	27.29 (1.2)
Middle (1.85–3.5)	33.01 (3.32)	26.87 (0.74)
High (>3.5)	33.68 (3.16)	37.53 (1.27)
Missing	6.84 (1.46)	8.31 (0.51)

a Data came from NHANES 1999–2004 only.

b Data came from NHANES III only.

DXA: dual energy X ray absorptiometry. BIA: tetrapolar bioelectrical impedance analysis.

All analyses were accounted for the complex sampling of the NHANES. N refers to the weighted sample size.

**Table 2:** Association between each of the obesity markers and the risk of developing all type MGUS.

Obesity Marker	Multivariable-adjusted ORs (95 % CI)
BMI (continuous), kg/m^2^	1.02 (0.99, 1.05)
BMI categories	
Normal: 18.5–25	Ref.
Underweight: ≤ 18.5	1.45 (0.52, 4.01)
Overweight: 25.0 to < 30	1.26 (0.93, 1.71)
Obese: ≥ 30	1.42 (0.98, 2.04)
Maximum lifetime BMI^a^	1.02 (0.99, 1.06)
Waist circumference (continuous), cm	1.01 (0.99, 1.02)
Waist circumference categories	
Normal risk (men, ≤102 cm; women, ≤88 cm)	Ref.
High risk (men, >102 cm; women, >88 cm)	1.06 (0.77, 1.46)
Waist to hip ratio^b^	1.01 (0.99, 1.02)
Fat-free mass (DXA)^a^, kg	1.00 (0.97, 1.02)
Fat-free mass (BIA)^b^, kg	1.01 (0.99, 1.04)
Total body fat (DXA)^a^, kg	1.02 (1.00, 1.04)
Total body fat (BIA)^b^, kg	1.01 (0.98, 1.03)
Body percentage fat (DXA)^a^, %	**1.04 (1.01, 1.07)**
Body percentage fat (BIA)^b^, %	1.00 (0.96, 1.04)

a Data from NHANES 1999–2004 only.

b Data from NHANES III only.

All logistic regression models were adjusted for demographic characteristics (i.e., age, gender, race, education level, and ratio of family income to poverty). All analyses were accounted for the complex sampling of the NHANES.

DXA: dual energy X ray absorptiometry. BIA: tetrapolar bioelectrical impedance analysis.

**Table 3:** Association between each of the obesity markers and the risk of developing IgG/IgA MGUS.

Obesity Markers	Multivariable-adjusted ORs (95 % CI)
BMI (continuous), kg/m^2^	1.02 (0.99, 1.06)
BMI categories	
Normal: 18.5–25	Ref.
Underweight: ≤ 18.5	1.68 (0.56, 5.11)
Overweight: 25.0 to < 30	1.11 (0.75, 1.66)
Obese: ≥ 30	1.49 (0.97, 2.31)
Maximum lifetime BMI^a^	1.02 (0.98, 1.06)
Waist (continuous), cm	1.01 (0.99, 1.02)
Waist categories	
Normal risk (men, ≤102 cm; women, ≤ 88 cm)	Ref.
High risk (men, >102 cm; women, >88 cm)	1.01 (0.70, 1.45)
Waist to hip ratio^b^	1.00 (0.98, 1.02)
Fat-free mass (DXA)^a^, kg	1.00 (0.98, 1.03)
Fat-free mass (BIA)^b^, kg	1.01 (0.98, 1.04)
Total body fat (DXA)^a^, kg	**1.02 (1.00, 1.05)**
Total body fat (BIA)^b^, kg	1.01 (0.98, 1.04)
Body percentage fat (DXA)^a^, %	**1.06 (1.02, 1.10)**
Body percentage fat (BIA)^b^, %	0.99 (0.95, 1.04)

a Data came from NHANES 1999–2004 only.

b Data came from NHANES III only.

All logistic regression models were adjusted for demographic characteristics (i.e., age, gender, race, education level, and ratio of family income to poverty). All analyses were accounted for the complex sampling of the NHANES.

DXA: dual energy X ray absorptiometry. BIA: tetrapolar bioelectrical impedance analysis

## Appendix A. Supporting information

Supplementary data associated with this article can be found in the online version at doi:10.1016/j.canep.2026.103021.
